# Metallothionein 1E mRNA is highly expressed in oestrogen receptor-negative human invasive ductal breast cancer

**DOI:** 10.1054/bjoc.2000.1276

**Published:** 2000-07-03

**Authors:** R Jin, B H Bay, V T K Chow, P H Tan, V C L Lin

**Affiliations:** Departments of Anatomy, Microbiology, Faculty of Medicine, National University of Singapore, Kent Ridge, S119260; Departments of Pathology, Clinical Research, Singapore General Hospital, Outram Road, S169608, Republic of Singapore

**Keywords:** breast cancer, metallothionein 1E isoform, RT-PCR, oestrogen receptor, progesterone receptor

## Abstract

Metallothioneins (MTs), a group of ubiquitous metalloproteins, comprise isoforms encoded by ten functional genes in humans. Different MT isoforms possibly play different functional roles during development or under various physiological conditions. The MT-1E isoform mRNA has been recently shown to be differentially expressed in oestrogen receptor (OR)-positive and OR-negative breast cancer cell lines. In this study, we evaluated MT-1E mRNA expression via semi-quantitative RT-PCR in 51 primary invasive ductal breast cancer tissues, concurrently with OR-positive and progesterone receptor (PR)-positive MCF7 cells, OR-negative and PR-negative MDA-MB-231 cells and PR-transfected MDA-MB-231 breast cancer cells (ABC28). We demonstrated significantly higher MT-1E mRNA expression in OR-negative compared with OR-positive breast cancer tissues (*P*= 0.026). MCF7 cells lacked MT-1E mRNA expression, while both OR- and PR-negative MDA-MD-231 cells exhibited a high level of MT-1E mRNA expression. The level of MT-1E mRNA expression in progesterone-treated and -untreated ABC28 cells remained similar as the parental cell line MDA-MB-231-C2 cells. The results suggest that MT-1E may have specific and functional roles in OR-negative invasive ductal breast cancers, possibly mediated via effector genes downstream of the oestrogen receptor, but not through the PR pathway. © 2000 Cancer Research Campaign


British Journal of Cancer (2000) **83**(3), 319–323
© 2000 Cancer Research Campaign
doi: 10.1054/ bjoc.2000.1276, available online at http://www.idealibrary.com on IDEAL®

### Metallothionein 1E mRNA is highly expressed in oestrogen receptor-negative human invasive ductal breast cancer

**R Jin[1], BH Bay[1], VTK Chow[2], PH Tan[3] and VCL Lin[4]**

[1]Departments of Anatomy and [2]Microbiology, Faculty of Medicine, National University of Singapore, Kent Ridge, S119260; Departments of [3]Pathology and [4]Clinical Research, Singapore General Hospital, Outram Road, S169608, Republic of Singapore

**Summary** Metallothioneins (MTs), a group of ubiquitous metalloproteins, comprise isoforms encoded by ten functional genes in humans. Different MT isoforms possibly play different functional roles during development or under various physiological conditions. The MT-1E isoform mRNA has been recently shown to be differentially expressed in oestrogen receptor (OR)-positive and OR-negative breast cancer cell lines. In this study, we evaluated MT-1E mRNA expression via semi-quantitative RT-PCR in 51 primary invasive ductal breast cancer tissues, concurrently with OR-positive and progesterone receptor (PR)-positive MCF7 cells, OR-negative and PR-negative MDA-MB-231 cells and PR-transfected MDA-MB-231 breast cancer cells (ABC28). We demonstrated significantly higher MT-1E mRNA expression in OR-negative compared with OR-positive breast cancer tissues ($P = 0.026$). MCF7 cells lacked MT-1E mRNA expression, while both OR- and PR-negative MDA-MD-231 cells exhibited a high level of MT-1E mRNA expression. The level of MT-1E mRNA expression in progesterone-treated and -untreated ABC28 cells remained similar as the parental cell line MDA-MB-231-C2 cells. The results suggest that MT-1E may have specific and functional roles in OR-negative invasive ductal breast cancers, possibly mediated via effector genes downstream of the oestrogen receptor, but not through the PR pathway. © 2000 Cancer Research Campaign

**Keywords**: breast cancer; metallothionein 1E isoform; RT-PCR; oestrogen receptor; progesterone receptor

Metallothioneins (MTs) are a group of ubiquitous low molecular mass, cysteine-rich intracellular metal-binding proteins (Kagi and Schaffer, 1988). MTs are known to play putative roles in cancer cell proliferation and apoptosis (Abdel-Mageed and Agrawal, 1997; Jayasurya et al, 2000) as well as in resistance to radiation and chemotherapeutic agents (Lohrer and Robson, 1989; Yang et al, 1994, Kondo et al, 1995). In breast cancer, MT overexpression has been well documented by immunohistochemical analysis (Schmid et al, 1993; Fresno et al, 1993; Haerslev et al, 1995; Goulding et al, 1995; Jin et al, 1999), and shown to be predominantly associated with poor prognosis.

In humans, the MTs are encoded by a family of genes consisting of 10 functional MT isoforms and the encoded proteins are conventionally subdivided into four groups viz, MT-1, MT-2, MT-3 and MT-4 proteins (Palmiter et al, 1992; Stennard et al, 1994; Quaife et al, 1994; Mididoddi et al, 1996). While a single MT-2A gene encodes MT-2 protein, MT-1 protein comprises many subtypes encoded by a set of MT-1 genes (MT-1A, MT 1-B, MT-1E, MT-1F, MT-1G, MT-1H and MT-1X) accounting for the microheterogeneity of the MT-1 protein (Karin et al, 1984). Different MT genes in humans possibly play different functional roles during development or under various physiological conditions (Kagi and Schaffer, 1988).

Differential expression of the MT-1E gene in oestrogen receptor (OR)-positive and -negative human breast cancer cell lines has been recently reported (Friedline et al, 1998). In that study, two OR-positive breast cancer cell lines (MCF7 and T-47D) showed no detectable MT-1E gene expression, whereas two OR-negative cell lines (MDA-MB-231 and Hs578T) expressed high levels of MT-1E mRNA. However, to the best of our knowledge, there is hitherto no available data on MT-1E mRNA expression in clinical breast cancer tissues, as most of published reports are on cell lines.

In light of the above findings, the aims of this study were to: a) analyse MT-1E mRNA expression in human invasive ductal breast cancer tissues concurrently with MCF7 and MDA-MB-231 human breast cancer cells; b) evaluate the association between MT-1E mRNA expression and the OR status of breast cancer tissues and; c) explore the relationship between MT-1E mRNA expression and PR status using PR-transfected MDA-MB-231 cell lines and breast cancer tissues.

### MATERIALS AND METHODS

### Human breast cancer tissues

Fifty-one primary invasive ductal breast cancer tissues were randomly obtained from mastectomies at the Singapore General Hospital (SGH). The diagnoses of invasive ductal breast cancer were made according to standard criteria defined by the World Health Organization (Elston and Ellis, 1998). None of the cases received chemotherapy or radiotherapy before surgery. The tissue samples were harvested by pathologists who divided each sample into two parts. One part was fixed in 10% formalin and embedded in paraffin for routine diagnosis by haematoxylin and eosin

*Received 23 December 1999*
*Revised 29 March 2000*
*Accepted 10 April 2000*

*Correspondence to:* BH Bay staining, and immunohistochemical assessment of OR and PR status. The other part, which was determined macroscopically as comprising only tumour tissue, was snap-frozen and stored in liquid nitrogen for mRNA analysis.

### Cell culture

All cells were routinely maintained in phenol-red containing Dulbecco's modified Eagle medium (DMEM) supplemented with 5% foetal calf serum (FCS), 2 mM glutamine and 40 mg $l^{-1}$ gentamicin. For all experiments, cells were grown in phenol-red-free DMEM supplemented with 5% dextran charcoal-treated fetal calf serum (DCC-FCS) to remove endogenous steroid hormones that might interfere with the analysis. Cells were treated with progesterone from a 1000-fold stock in ethanol, giving a final ethanol concentration of 0.1%. Treatment controls received 0.1% ethanol only. MCF-7 (OR+, PR+) and MDA-MB-231 (OR–, PR–) cells were obtained from the American Type Culture Collection.

### Transfection

Transfection of PR cDNA into OR- and PR-negative MDA-MB-231 breast cancer cells is as described previously (Lin et al, 1999). PR expression vectors hPR1 and hPR2 were generous gifts from Professor Chambon P (IGBMC, France). Vectors hPR1 and hPR2 contained human PR cDNA encoding PR isoform B and isoform A, respectively, in pSG5 plasmid. Vector pBK-CMV (Stratagene, La Jolla, USA) containing the neomycin-resistant gene was co-transfected with hPR1 and hPR2 into MDA-MB-231-CL2 cells. Neomycin-resistant clones were screened for PR using the PR enzyme immunoassay (EIA) kit (Abbott Laboratories, Illinois, USA). Eight PR-positive clones expressing both isoforms A and B were isolated and characterized. They showed similar responses to progesterone treatment (Lin et al, 1999). The effects of progesterone on MT-1E mRNA expression in clone ABC28 that expressed ~660 fmol PR per mg protein were studied in this report. Cells stably transfected with both vectors pBK-CMV and pSG5 (clone pSG5-c15) served as control transfectant.

### RT-PCR analysis

Total RNA was isolated using the RNeasy Mini kit (QIAGEN, Hilden, Germany) according to the manufacturer's protocol. The concentration and purity of the extracted RNA were evaluated by spectrophotometric absorbance readings at 260 and 280 nm.

Total RNA was reverse-transcribed using Superscript II RNase $H^-$ reverse transcriptase (Life Technologies, Gaithersburg, USA) in 1 × RT buffer (50 mM Tris-HCl pH 8.3, 75 mM KCl, 3 mM $MgCl_2$), 0.01 M DTT, 0.5 U $\mu l^{-1}$ RNase inhibitor, 0.33 mM of each dNTP, and 0.003 $OD_{260}$ U $\mu l^{-1}$ random primers (Life Technologies). The samples were incubated at room temperature for 10 min, then at 42°C for 60 min, followed by 5 min at 99°C, and at 4°C for 5 min. Each cDNA sample equivalent to about 80 ng total RNA was then used for PCR amplification. Twenty-five PCR cycles were used, each consisting of denaturation for 30 s at 95°C, annealing for 30 s at 65°C, and extension for 30 s at 72°C. The initial step was 95°C for 1 min, with a final elongation step of 72°C for 7 min. The PCR primers used were as described by Mididoddi et al (1996). The resultant PCR products were electrophoresed in 1.6% agarose gels containing ethidium bromide along with DNA markers, visualized under UV, and analysed by densitometric scanning (Bio-Rad model GS-700 Imaging Densitometer) using the Molecular Analyst Software (Bio-Rad Laboratories version 1.5). Two to three independent PCRs were performed to control for variations between experiments. A no-template control in which water was added instead of RNA and a no-reverse transcriptase control in which water was added instead of the enzyme were included for each PCR batch. For semi-quantitative analysis, mRNA of the housekeeping gene glyceraldehyde-3-phosphate dehydrogenase (G3PDH) was co-amplified under the same conditions using the amplimers described by Abdel-Mageed et al (1997). The relative expression level of each MT isoform compared to G3PDH gene expression was determined. A prior PCR cycle optimization was conducted to ensure that all the reactions remained in the linear region (by removal and analysis of samples at 15, 20, 25, 30 and 35 cycles). A selected pre-experimental RT-PCR product was proven to be actual MT-1E isoforms by cycle dideoxy DNA sequencing, and several representative PCR products were reconfirmed by restriction endonuclease digestion analysis.

### Restriction endonuclease digestion of MT-1E RT-PCR products

Following RT-PCR, 15 µl of PCR product was subjected to restriction enzyme digestion by adding 2 µl of relevant buffer, 10 units Bgl-I or Ear-I enzyme (New England BioLabs, Beverly, MA, USA) with 3 µl distilled water to a final volume of 20 µl. Following incubation at 37°C for 1 h, the reaction was terminated by adding 5 µl gel-loading buffer, samples were electrophoresed in 1.6% agarose gels along with DNA markers and visualized under UV. Based on the specific sequence in the 5′ and 3′ untranslated regions of the active MT-1E gene (Mididoddi et al, 1996), the MT-1E primers can amplify a 284-bp RT-PCR product with a Bgl-I site that would give rise to two fragments (164 bp and 120 bp). Two Ear-I sites within the target amplicon would be expected to result in three fragments (137 bp, 108 bp and 39 bp), provided the reaction was also specific for the MT-1E gene.

### Immunohistochemistry

OR and PR status were respectively determined by immunohistochemistry. 3 µm sections were cut from each block, mounted on slides coated with 'APES' (3-aminopropyltriethoxysilane; purchased from Sigma; St. Louis, MO, USA) and dried overnight at 55°C. The sections were deparaffinized and rehydrated. Endogenous peroxidase activity was blocked by dipping the slides in 12 ml of methanol containing 200 µl hydrogen peroxide for 10 min followed by washing in tap water. The slides were then placed in 0.01 M citrate buffer (pH 6.0) and pressure cooked for 2 min before washing in tap water and rinsing with Tris buffered saline (TBS) at pH 7.6. The primary antibody was then applied. The oestrogen receptor primary antibody used was ERID5 clone (Dako Copenhagen, Denmark), at 1:30 dilution. The progesterone receptor primary antibody used was PRIA6 clone (Dako) at 1:30 dilution. The primary antibody was incubated for 1 h before rinsing off with TBS. This was followed by incubation with biotinylated anti-mouse immunoglobulin for 20 min, after which the sections were incubated with streptavidin–biotin complex for another 20 min. Freshly prepared DAB (5 mg 3,3-diaminoazobenzidine

 © 2000 Cancer Research Campaign tetrahydrochloride; Sigma), dissolved in 10 ml Tris/HCL buffer at pH 7.6 containing 100 µl of 1% hydrogen peroxide, was applied for 10 min after the tertiary layer was rinsed off with TBS. DAB was removed by rinsing with TBS and the slides were counterstained with haematoxylin. They were then differentiated in 0.5% acid alcohol and placed under running water. The final steps were dehydration, clearing and mounting in depex. Positive OR and PR staining was defined when 10% or more of tumour cell nuclei were immunoreactive.

### Statistical analysis

The Graphpad Prism software package was used for statistical analysis. Non-parametric Mann–Whitney test was performed to compare sample medians. Pearson's correlation was used to analyse the relationship between MT-1E mRNA expression with age and tumour size.

### RESULTS

### MT-1E mRNA expression and specificity

Except for one OR-positive breast cancer sample, the mRNA of the MT-1E isoform was detected via RT-PCR in the other 50 cancerous samples (Figure 1). The mean ratio of MT-1E expression (relative to housekeeping gene G3PDH) was 0.52 ± 0.04 (± sem). The frequency histogram is shown in Figure 2. One OR-negative breast cancer sample had a very high level of MT-1E mRNA expression (ratio was 1.99). OR- and PR-positive MCF7 cells showed no detectable MT-1E mRNA expression, whereas OR- and PR-negative MDA-MB-231 cells exhibited high levels of MT-1E mRNA expression (Figure 3).

The specificity of the MT-1E RT-PCR products was confirmed by sequencing and restriction enzyme digestion of representative RT-PCR products. Restriction enzyme digestion of MT-1E RT-PCR product is exemplified in Figure 4.

### MT-1E mRNA expression and steroid receptor status

As evaluated by immunohistochemistry, 35 samples were OR-positive and 16 cases were OR-negative, while 17 cases were PR-positive with 34 PR-negative cases (Table 1).

High MT-1E mRNA expression in OR-negative tumour compared with OR-positive tumours was statistically significant (0.68 ± 0.10 vs 0.45 ± 0.04, $P$ = 0.026). There was no significant difference of MT-1E expression between PR-negative and PR-positive breast tumours (0.54 ± 0.058 vs 0.46 ± 0.053, $P$ = 0.43), nor was there a significant difference between PR-positive and PR-negative tumours in the OR-positive subgroup (0.46 ± 0.053 vs 0.44 ± 0.053, $P$ = 0.79) (Table 1).

In order to confirm whether MT-1E expression is affected by PR expression and progesterone treatment, we examined MT-1E mRNA levels in PR-transfected MDA-MB-231 breast cancer cells clone ABC28 and in untransfected controls. PR transfectant ABC28 cells expressed similar levels of MT-1E mRNA as the OR- and PR-negative control transfectant pSG5–C15 and the parental cell line MDA-MB-231-C2 (Figure 3). Treatment of ABC28 cells with 0.1 µM Progesterone for 24 h had no effect on MT-1E expression compared with vehicle-treated controls (Figure 3, lanes 6 and 7). To further validate the results, we tested the effect of progesterone treatment on MT-1E expression in ABC28 cells at a series of time points (data not shown). Again, there was no difference in MT-1E gene expression after 3, 6, 9 and 24 h of treatment between progesterone-treated and vehicle-treated control ABC28 cells which had been shown to respond to progesterone treatment in a number of cellular processes such as cellular proliferation and focal adhesion (Lin et al, 1999; 2000).

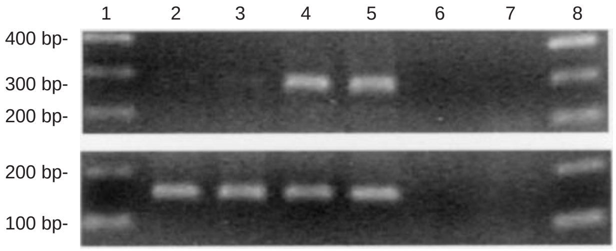

**Figure 1** Example of MT-IE mRNA expression (reflected by 284 bp RT-PCR products) in invasive ductal carcinomas. Top panel: DNA markers (lanes 1 and 8), OR-positive cancer tissue from the same sample (lanes 2 and 3), OR-negative cancer tissue (lanes 4 and 5), 'no RNA' control (lane 6) and 'no-reverse transcript' control. Bottom panel: housekeeping gene G3PDH expression (160 bp RT-PCR product) corresponding to lanes in top panel

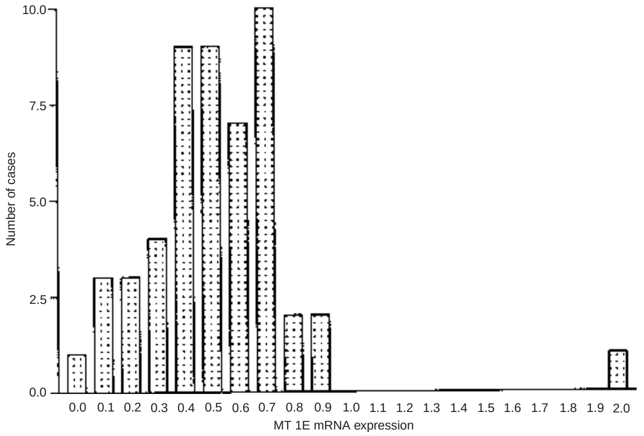

**Figure 2** Frequency histogram of MT-1E mRNA expression level (relative to expression of G3PDH) in breast cancer tissues

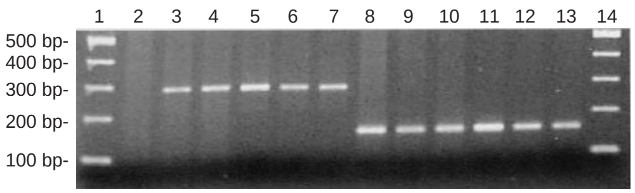

**Figure 3** Agarose gel electrophoresis showing MT-1E mRNA expression in breast cancer cell lines. DNA marker (lanes 1 and 14), MCF7 (lane 2), MDA-MB-231 (lane 3), ABC28 cells (lane 4), pSG5-C15 cells (lane 5), ABC28 cells under 24 h of progesterone treatment (lane 6) and 0.1% ethanol treatment (lane 7). Lanes 8 to 13 show housekeeping gene G3PDH expression corresponding to lanes 2–7, respectively.

© 2000 Cancer Research Campaign

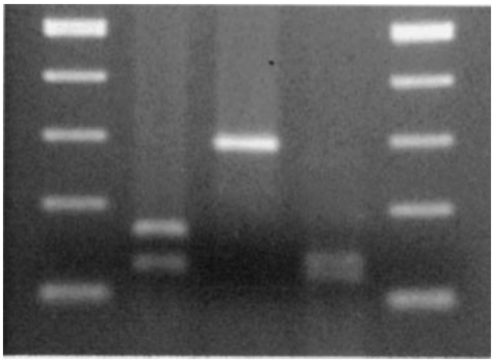

**Figure 4** Agarose gel electrophoresis showing restriction enzyme cleavage of MT-1E RT-PCR products. DNA marker (lanes 1 and 5); bgl-I digested product (lane 2), showing expected 164 bp and 120 bp fragments; undigested 284 bp product (lane 3); Ear-I digested product (lane 4), showing 137 bp and 108 bp fragments (39 bp fragment not shown).

No significant associations were observed between MT-1E mRNA expression and other clinicopathological factors, such as patient age, tumour size and histological grade.

### DISCUSSION

Physical mapping studies have shown that all known functional human MT-1 and MT-2 genes are clustered on chromosome 16*q13*. MT isoforms display specific developmental expression patterns (Andrews et al, 1991) as well as cell-type-specific and inducer-specific regulation (Richards et al, 1984; Schmidt and Hamer, 1986; Jahroudi et al, 1990; Cavigelli et al, 1993). In addition, MT primary sequences are highly conserved among vertebrates. It is therefore not surprising that members of the MT family may be involved pleiotropically in a number of different biological functions (Kagi and Schaffer, 1988).

Since the coding regions of MT isoforms are highly conserved, experiments on any individual isoform should be very carefully conducted to ensure that the exact isoform is analysed. In this study, we authenticated actual MT-1E gene amplification by gene sequencing and restriction enzyme digestion analyses. Our results showing no detectable MT-1E expression in OR-positive MCF 7 breast cancer cells and high levels of MT-1E expression in the OR-negative breast cancer cell line MDA-MB-231 concurs with the finding of Friedline and co-workers (1998). Furthermore, we demonstrated significantly higher MT-1E mRNA expression levels in OR-negative than in OR-positive human breast cancer tissues. It is also interesting to note that one of the OR-negative breast cancer samples, had an outstandingly high level of MT-1E expression. This overexpression of MT-1E in this breast cancer tissue could be explained by the possibility of a gene mutation, as recently suggested by Jasani et al (1998).

Oestrogen receptor status is an important prognostic marker of breast cancer. OR-negative tumours generally incur a poor prognosis and are resistant to hormone therapy (Rich et al, 1978; McKenzie and Sukumar, 1996). Oestrogen and progesterone play central roles in normal breast development, as well as in breast carcinogenesis, by regulating the expression of a variety of genes through their receptors (Celentano et al, 1998). The oestrogen receptor is considered to be a ligand-activated transcription factor which upon oestrogen binding alters transcription of target genes,

**Table 1** Mean values of MT-1E mRNA expression (relative to housekeeping gene G3PDH) in human invasive ductal breast cancer tissues with OR and PR status

| | OR+ ($n$ = 35) | OR– ($n$ = 16) | Total ($n$ = 51) |
|---|---|---|---|
| PR+ ($n$ = 17) | 0.46±0.053 (17) | (0) | 0.46±0.053 (17) |
| PR– ($n$ = 34) | 0.44±0.053 (18) | 0.68±0.10 (16) | 0.54±0.058 (34) |
| Total ($n$ = 51) | 0.45±0.040 (35) | 0.68±0.10 (16) | 0.52±0.040 (51) |

Results are expressed as mean ± sem. Brackets indicate number of cases thereby affecting cellular processes such as mitogenesis (Murphy et al, 1998; Dowsett, 1998).

Transformation from an OR- and PR-positive hormone-dependent tumour to an OR- and PR-negative hormone-independent tumour is a crucial step in the progression of breast cancer. This process would require an alternative mechanism to replace the functions of oestrogen and progesterone (Mckenzie and Sukumar, 1996). It is possible that MT-1E may participate in that role. MTs are known to influence tumour growth and survival by promoting cell proliferation and cellular repair processes, enhancing resistance to chemotherapy and inhibiting apoptosis (Kagi 1991; Kondo et al, 1995; Abdel-Mageed and Agrawal, 1997; Jayasurya et al, 2000). High MT expression has also been reported to be associated with aggressive behaviour in endometrial carcinoma (McCluggage et al, 1999). The known biological functions of MT and the association of MT-1E with OR-breast cancer concurs with the observation that breast cancer patients who do not exhibit ORs have a poorer prognosis and decreased disease-free survival as compared with patients whose tumours contained ORs (Wittliff et al, 1998).

Progesterone receptors have a close relationship with OR and are known to be regulated by oestrogens (Rayter, 1991). However, we found no significant difference between PR-negative and PR-positive breast cancer tissues. In OR-positive breast cancer tissues, there is no difference whether the tissue is also PR-positive or PR-negative. Our findings that PR-transfected (ABC28) and untransfected (MDA-MB-231) cells expressed similar levels of MT-1E mRNA, and that progesterone treatment of ABC28 did not down-regulate MT-1E mRNA expression suggest that the role of MT-1E in OR-negative breast cancers is not mediated via the PR pathway.

In conclusion, we have demonstrated a higher MT-1E mRNA expression in OR-negative than in OR-positive invasive ductal breast cancer tissues which is independent of the PR status. Whether MT-1E expression influences the poor prognosis associated with OR-negative breast tumours remains to be determined. It is therefore worthwhile to extend studies on the role of MT-1E in breast cancers by adopting molecular approaches.

### ACKNOWLEDGEMENTS

We thank Professor P Chambon from the Institute of Genetics and Molecular and Cellular Biology, Strasbourg, France, for kindly providing the expression vectors hPR1 and hPR2. We are grateful to WM Yeo and TY Yick for technical assistance and the Department of Pathology, Singapore General Hospital and National Cancer Centre for tissue collection. This research was supported by a grant (NMRC/0244/1997) from the National Medical Research Council, Singapore.

© 2000 Cancer Research Campaign

### REFERENCES

Abdel-Mageed AB and Agrawal KC (1997) Antisense down-regulation of metallothionein induces growth arrest and apoptosis in human breast carcinoma cells. *Cancer Gene Ther* **4**: 199–207

Andrews GK, Huet-Hudson YM, Paria BC, McMaster MT, De SK and Dey SK (1991) Metallothionein gene expression and metal regulation during preimplantation mouse embryo development (MT mRNA during early development). *Dev Biol* **145**: 13–27

Cavigelli M, Kagi JHR, and Hunziker PE (1993) Cell- and inducer-specific accretion of human isometallothioneins. *Biochem J* **292**: 551–554

Celentano E, Montella M, Bonelli P, Cecco L, Marco MD, Botti G and D'Aiuto G (1998) Does a relationship exist between trends in oestrogen receptor levels and breast cancer incidence and mortality? *Int J Oncol* **13**: 129–135

Dowsett M (1998) Improved prognosis for biomarkers in breast cancer. *Lancet* **351**: 1753–1754

Elston CW and Ellis IO (1998) *Systemic Pathology, Vol 13, The Breast, 3rd edn.* Churchill Livingstone: Edinburgh

Fresno M, Wu W, Rodriguez JM and Nadji M (1993) Localization of metallothionein in breast carcinomas. An immunohistochemical study. *Virchows Archiv A Pathol Anat Histopathol* **423**: 215–219

Friedline JA, Garrett SH, Somji S, Todd JH and Sens DA (1998) Differential expression of the MT-1E gene in oestrogen-receptor-positive and -negative human breast cancer cell lines. *Am J Pathol* **152**: 23–27

Goulding H, Jasani B, Pereira H, Reid A, Galea M, Bell JA, Elston CW, Robertson JF, Blamey RW, Nicholson RA, Schmid KW and Ellis IO (1995) Metallothionein expression in human breast cancer. *Br J Cancer* **72**: 968–972

Haerslev T, Jacobsen GK and Zedeler (1995) The prognostic significance of immunohistochemically detectable metallothionein in primary breast carcinomas. *APMIS* **103**: 279–285

Jahroudi N, Foster R, Price-Haughey J, Beitel G and Gedamu L (1990) Cell-type specific and differential regulation of the human metallothionein genes. *J Biol Chem* **265**: 6506–6511

Jasani B, Campbell F, Navabi H, Schmid KW and Williams GT (1998) Clonal overexpression of metallothionein is induced by somatic mutation in morphologically normal colonic mucosa. *J Pathol* **184**: 144–147

Jayasurya A, Bay BH, Yap WM and Tan NG (2000) Correlation of metallothionein expression with apoptosis in nasopharyngeal carcinoma. *Br J Cancer*: **82**: 1198–1203

Jin R, Bay B, Tan P and Tan BK (1999) Metallothionein expression and zinc levels in invasive ductal breast carcinoma. *Oncol Rep* **6**: 871–875

Kagi JHR and Schaffer A (1988) Biochemistry of metallothionein. *Biochemistry* **27**: 8509–8515

Kagi JHR (1991) Overview of metallothioneins. *Methods Enzymol* **205**: 613–626

Karin M, Eddy RL, Henry WM, Haley LL, Byers MG and Shows TB (1984) Human metallothionein genes are clustered on chromosome 16. *Proc Natl Acad Sci USA* **81**: 5494–5498

Kondo Y, Kuo SM, Watkins SC and Lazo JS (1995) Metallothionein localization and cisplatin resistance in human hormone-independent prostatic tumour cell lines. *Cancer Res* **55**: 474–477

Lin VCL, Ng EH, Aw SE, Tan MGK, Ng EHL, Chan VSW and Ho GH (1999) Progestins inhibit the growth of MDA-MB-231 cells transfected with progesterone receptor complementary DNA. *Clin Cancer Res* **5**: 395–403

Lin VCL, Ng EH, Aw SE, Tan MGK, Ng EHL and Bay BH (2000) Progesterone induces focal adhesion in breast cancer cells MDA-MB-231 transfected with progesterone receptor cDNA. *Mol Endocrinol* **14**: 348–358

Lohrer H and Robson T (1989) Overexpression of metallothionein in CHO cells and its effect on cell killing by ionizing radiation and alkylating agents. *Carcinogenesis* **10**: 2279–2284

McCluggage WG, Maxwell P, Hamilton PW and Jasani B (1999) High metallothionein expression is associated with features predictive of aggrfessive behaviour in endometrial carcinoma. *Histopathology* **34**: 51–55

Mckenzie K and Sukumar S (1996) Molecular genetics of human breast cancer. In *Cellular and Molecular Mechanisms of Hormonal Carcinogenesis*, Huff J, Boyd J and Barrett JC (eds), pp. 183–209. Wiley-Liss: New York

Mididoddi S, McGuirt JP, Sens MA, Todd JH and Sens DA (1996) Isoform-specific expression of metallothionein mRNA in the developing and adult human kidney. *Toxicol Lett* **685**: 17–27

Murphy LC, Dotzlaw H, Leygue E, Coutts A and Watson P (1998) The pathophysiological role of oestrogen receptor variants in human breast cancer. *J Steroid Biochem Molec Biol* **65**: 175–180

Palmiter RD, Findley SD, Whitmore TE and Durnam DM (1992) MT-III, a brain-specific member of the metallothionein gene family. *Proc Natl Acad Sci USA* **89**: 6333–6337

Quaife CJ, Findley SD, Erickson JC, Froelick GJ, Kelly EJ, Zambrowicz BP and Palmiter RD (1994) Induction of a new metallothionein isoform (MT-IV) occurs during differentiation of stratified squamous epithelia. *Biochemistry* **33**: 7250–7259

Rayter Z (1991) Steroid receptors in breast cancer. *Br J Surg* **78**: 528–535

Richards RI, Heguy A and Karin M (1984) Structural and functional analysis of the human metallothionein-1A gene: differential induction by metal ions and glucocorticoids. *Cell* **37**: 263–272

Rich MA, Furmanski P and Brooks SC (1978) Prognostic value of oestrogen receptor determinations in patients with breast cancer. *Cancer Res* **38**: 4296–4298

Schmid KW, Ellis IO, Gee JMW, Darke BM, Lees WE, Kay J, Cryer A, Stark JM, Hittmair A, Ofner Dunser M, Margreiter R, Daxenhichler G, Nicholson RI, Bier B, Bocker W and Jasani B (1993) Presence and possible significance of immunocytochemically demonstrable metallothionein over-expression in primary invasive ductal carcinoma of the breast. *Virchows Archiv A Pathol Anat Histopathol* **422**: 153–159

Schmidt CJ and Hamer DH (1986) Cell specificity and an effect of ras on human metallothionein gene expression. *Proc Natl Acad Sci USA* **83**: 3346–3350

Stennard FA, Holloway AF, Hamilton J and West AK (1994) Characterization of six additional human metallothionein genes. *Biochim Biophys Acta* **1218**: 357–365

West AK, Stallings R, Hildebrand CE, Chiu R, Karin M and Richards RI (1990) Human metallothionein genes: structure of the functional locus at 16q13. *Genomics* **8**: 513–518

Wittliff JL, Pasic R and Bland KI (1998) Steroid and peptide hormone receptors: methods, quality control and clinical use. In *The Breast ,Vol 1*, Bland KI, Copeland EM (eds), pp. 458–498. WB Saunders Co: Philadelphia

Yang YY, Woo ES, Reese CE, Bahnson RR, Saijo N and Lazo JS (1994) Human metallothionein isoform gene expression in cisplatin-sensitive and resistant cells. *Mol Pharmacol* **45**: 453–460

*© 2000 Cancer Research Campaign*

## References

[PDF_00406] Abdel-Mageed A., Agrawal K. C. (1997). Antisense down-regulation of metallothionein induces growth arrest and apoptosis in human breast carcinoma cells.. Cancer Gene Ther.

[PDF_00409] Andrews G. K., Huet-Hudson Y. M., Paria B. C., McMaster M. T., De S. K., Dey S. K. (1991). Metallothionein gene expression and metal regulation during preimplantation mouse embryo development (MT mRNA during early development).. Dev Biol.

[PDF_00413] Cavigelli M., Kägi J. H., Hunziker P. E. (1993). Cell- and inducer-specific accretion of human isometallothioneins.. Biochem J.

[PDF_00415] Celentano E., Montella M., Bonelli P., Cecco L., De Marco M., Di Cintio P., Iannuzzo M., Tuccillo F., Botti G., D'Aiuto G. (1998). Does a relationship exist between trends in estrogen receptor levels and breast cancer incidence and mortality?. Int J Oncol.

[PDF_00418] Dowsett M. (1998). Improved prognosis for biomarkers in breast cancer.. Lancet.

[PDF_00422] Fresno M., Wu W., Rodriguez J. M., Nadji M. (1993). Localization of metallothionein in breast carcinomas. An immunohistochemical study.. Virchows Arch A Pathol Anat Histopathol.

[PDF_00425] Friedline J. A., Garrett S. H., Somji S., Todd J. H., Sens D. A. (1998). Differential expression of the MT-1E gene in estrogen-receptor-positive and -negative human breast cancer cell lines.. Am J Pathol.

[PDF_00428] Goulding H., Jasani B., Pereira H., Reid A., Galea M., Bell J. A., Elston C. W., Robertson J. F., Blamey R. W., Nicholson R. A. (1995). Metallothionein expression in human breast cancer.. Br J Cancer.

[PDF_00431] Haerslev T., Jacobsen G. K., Zedeler K. (1995). The prognostic significance of immunohistochemically detectable metallothionein in primary breast carcinomas.. APMIS.

[PDF_00434] Jahroudi N., Foster R., Price-Haughey J., Beitel G., Gedamu L. (1990). Cell-type specific and differential regulation of the human metallothionein genes. Correlation with DNA methylation and chromatin structure.. J Biol Chem.

[PDF_00437] Jasani B., Campbell F., Navabi H., Schmid K. W., Williams G. T. (1998). Clonal overexpression of metallothionein is induced by somatic mutation in morphologically normal colonic mucosa.. J Pathol.

[PDF_00440] Jayasurya A., Bay B. H., Yap W. M., Tan N. G. (2000). Correlation of metallothionein expression with apoptosis in nasopharyngeal carcinoma.. Br J Cancer.

[PDF_00443] Jin R., Bay B., Tan P., Tan B. K. (1999). Metallothionein expression and zinc levels in invasive ductal breast carcinoma.. Oncol Rep.

[PDF_00448] Karin M., Eddy R. L., Henry W. M., Haley L. L., Byers M. G., Shows T. B. (1984). Human metallothionein genes are clustered on chromosome 16.. Proc Natl Acad Sci U S A.

[PDF_00451] Kondo Y., Kuo S. M., Watkins S. C., Lazo J. S. (1995). Metallothionein localization and cisplatin resistance in human hormone-independent prostatic tumor cell lines.. Cancer Res.

[PDF_00447] Kägi J. H. (1991). Overview of metallothionein.. Methods Enzymol.

[PDF_00445] Kägi J. H., Schäffer A. (1988). Biochemistry of metallothionein.. Biochemistry.

[PDF_00457] Lin V. C., Ng E. H., Aw S. E., Tan M. G., Ng E. H., Bay B. H. (2000). Progesterone induces focal adhesion in breast cancer cells MDA-MB-231 transfected with progesterone receptor complementary DNA.. Mol Endocrinol.

[PDF_00454] Lin V. C., Ng E. H., Aw S. E., Tan M. G., Ng E. H., Chan V. S., Ho G. H. (1999). Progestins inhibit the growth of MDA-MB-231 cells transfected with progesterone receptor complementary DNA.. Clin Cancer Res.

[PDF_00460] Lohrer H., Robson T. (1989). Overexpression of metallothionein in CHO cells and its effect on cell killing by ionizing radiation and alkylating agents.. Carcinogenesis.

[PDF_00463] McCluggage W. G., Maxwell P., Hamilton P. W., Jasani B. (1999). High metallothionein expression is associated with features predictive of aggressive behaviour in endometrial carcinoma.. Histopathology.

[PDF_00469] Mididoddi S., McGuirt J. P., Sens M. A., Todd J. H., Sens D. A. (1996). Isoform-specific expression of metallothionein mRNA in the developing and adult human kidney.. Toxicol Lett.

[PDF_00472] Murphy L. C., Dotzlaw H., Leygue E., Coutts A., Watson P. (1998). The pathophysiological role of estrogen receptor variants in human breast cancer.. J Steroid Biochem Mol Biol.

[PDF_00475] Palmiter R. D., Findley S. D., Whitmore T. E., Durnam D. M. (1992). MT-III, a brain-specific member of the metallothionein gene family.. Proc Natl Acad Sci U S A.

[PDF_00478] Quaife C. J., Findley S. D., Erickson J. C., Froelick G. J., Kelly E. J., Zambrowicz B. P., Palmiter R. D. (1994). Induction of a new metallothionein isoform (MT-IV) occurs during differentiation of stratified squamous epithelia.. Biochemistry.

[PDF_00482] Rayter Z. (1991). Steroid receptors in breast cancer.. Br J Surg.

[PDF_00486] Rich M. A., Furmanski P., Brooks S. C. (1978). Prognostic value of estrogen receptor determinations in patients with breast cancer.. Cancer Res.

[PDF_00483] Richards R. I., Heguy A., Karin M. (1984). Structural and functional analysis of the human metallothionein-IA gene: differential induction by metal ions and glucocorticoids.. Cell.

[PDF_00489] Schmid K. W., Ellis I. O., Gee J. M., Darke B. M., Lees W. E., Kay J., Cryer A., Stark J. M., Hittmair A., Ofner D. (1993). Presence and possible significance of immunocytochemically demonstrable metallothionein over-expression in primary invasive ductal carcinoma of the breast.. Virchows Arch A Pathol Anat Histopathol.

[PDF_00495] Schmidt C. J., Hamer D. H. (1986). Cell specificity and an effect of ras on human metallothionein gene expression.. Proc Natl Acad Sci U S A.

[PDF_00498] Stennard F. A., Holloway A. F., Hamilton J., West A. K. (1994). Characterisation of six additional human metallothionein genes.. Biochim Biophys Acta.

[PDF_00501] West A. K., Stallings R., Hildebrand C. E., Chiu R., Karin M., Richards R. I. (1990). Human metallothionein genes: structure of the functional locus at 16q13.. Genomics.

[PDF_00507] Yang Y. Y., Woo E. S., Reese C. E., Bahnson R. R., Saijo N., Lazo J. S. (1994). Human metallothionein isoform gene expression in cisplatin-sensitive and resistant cells.. Mol Pharmacol.

